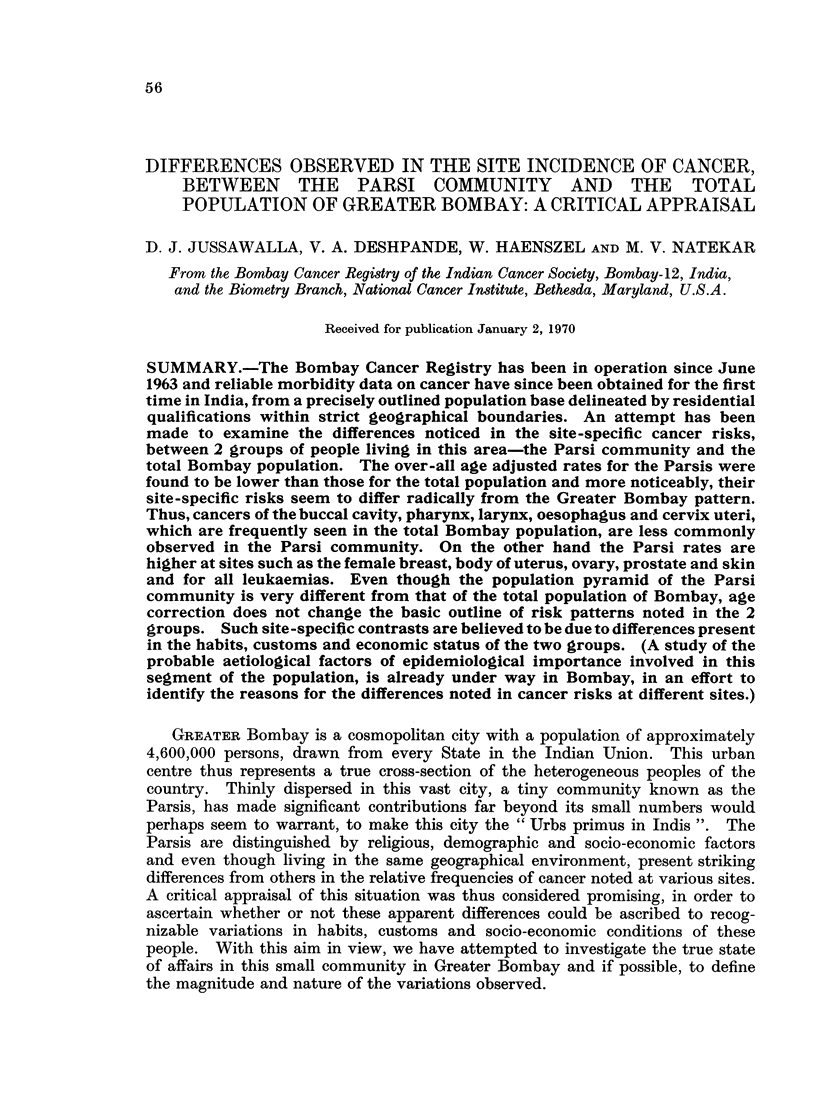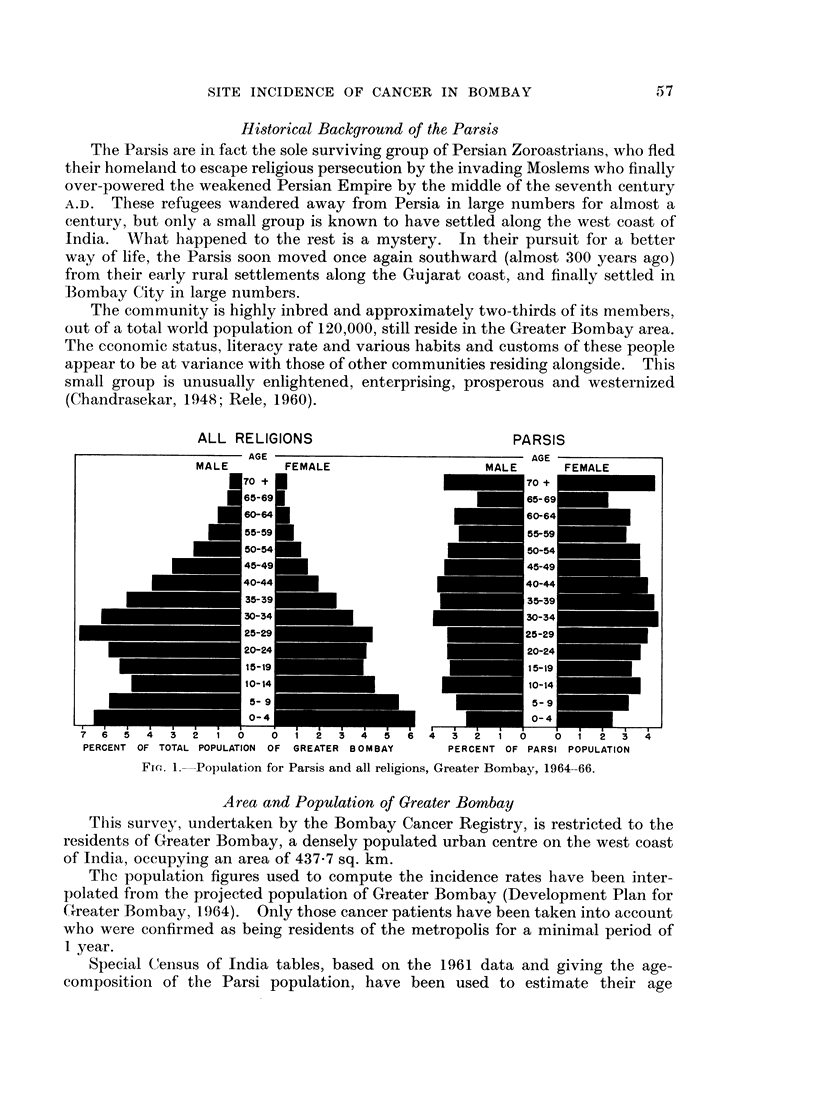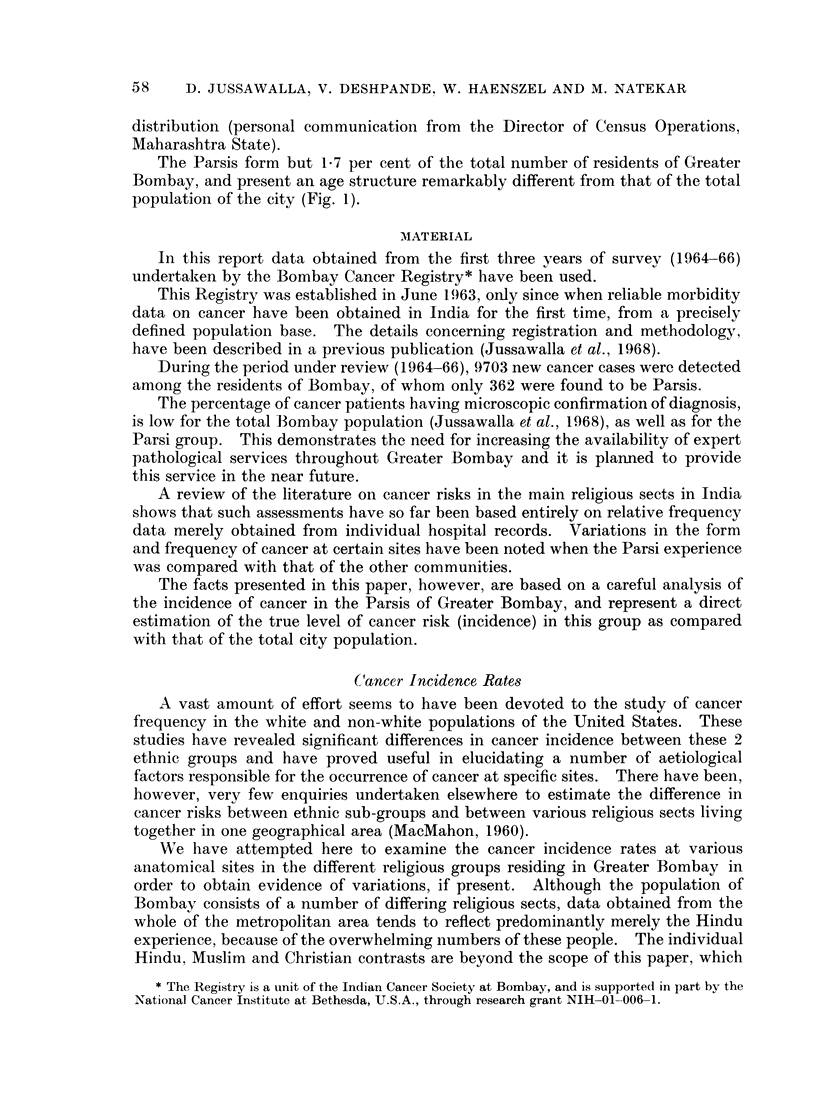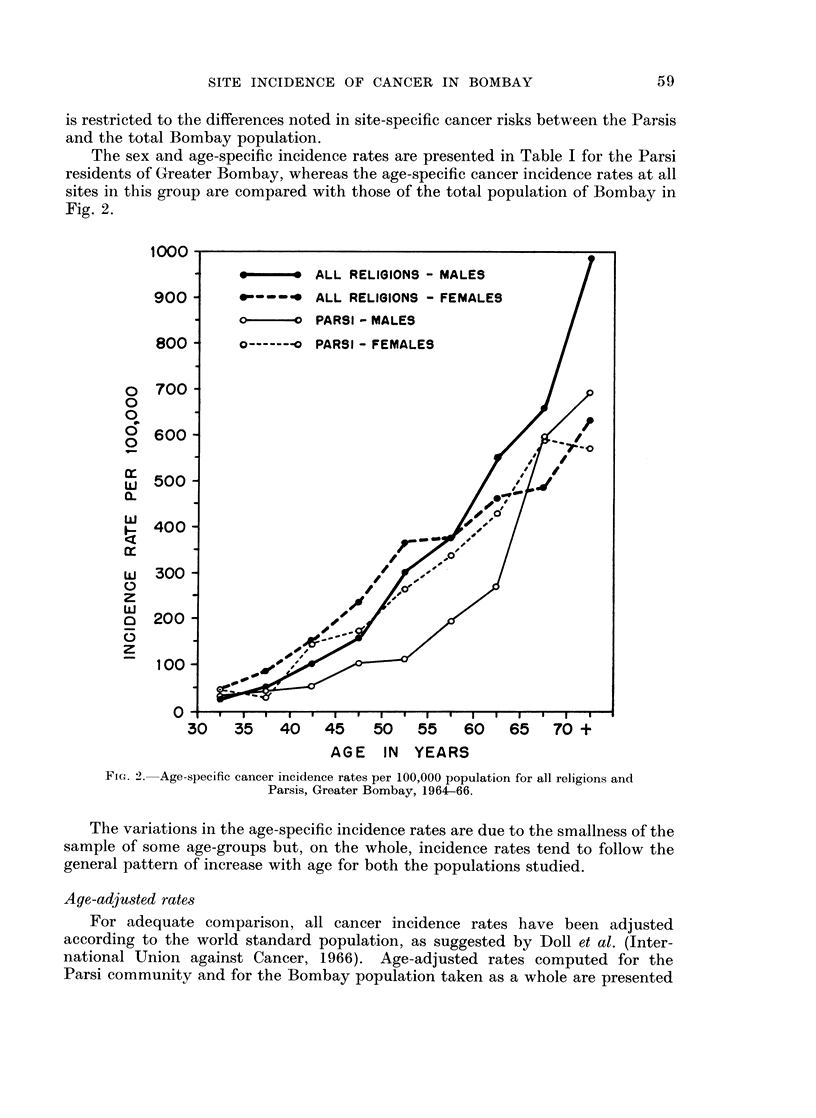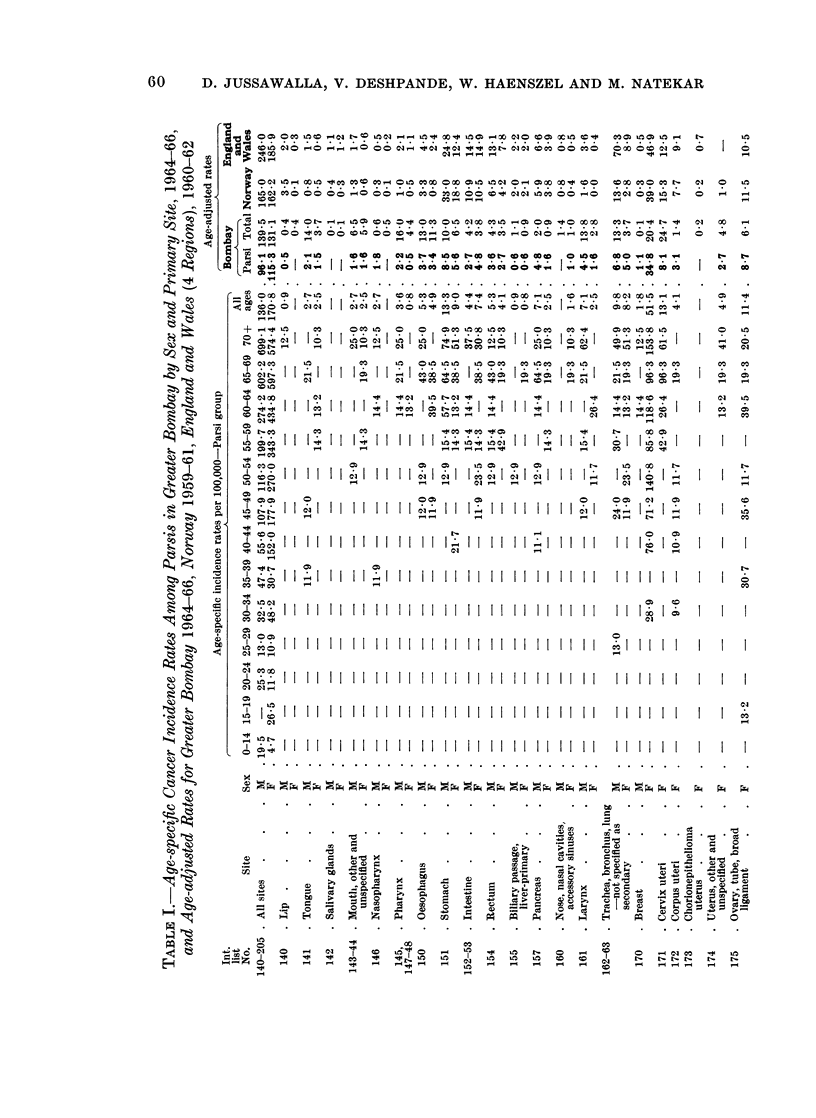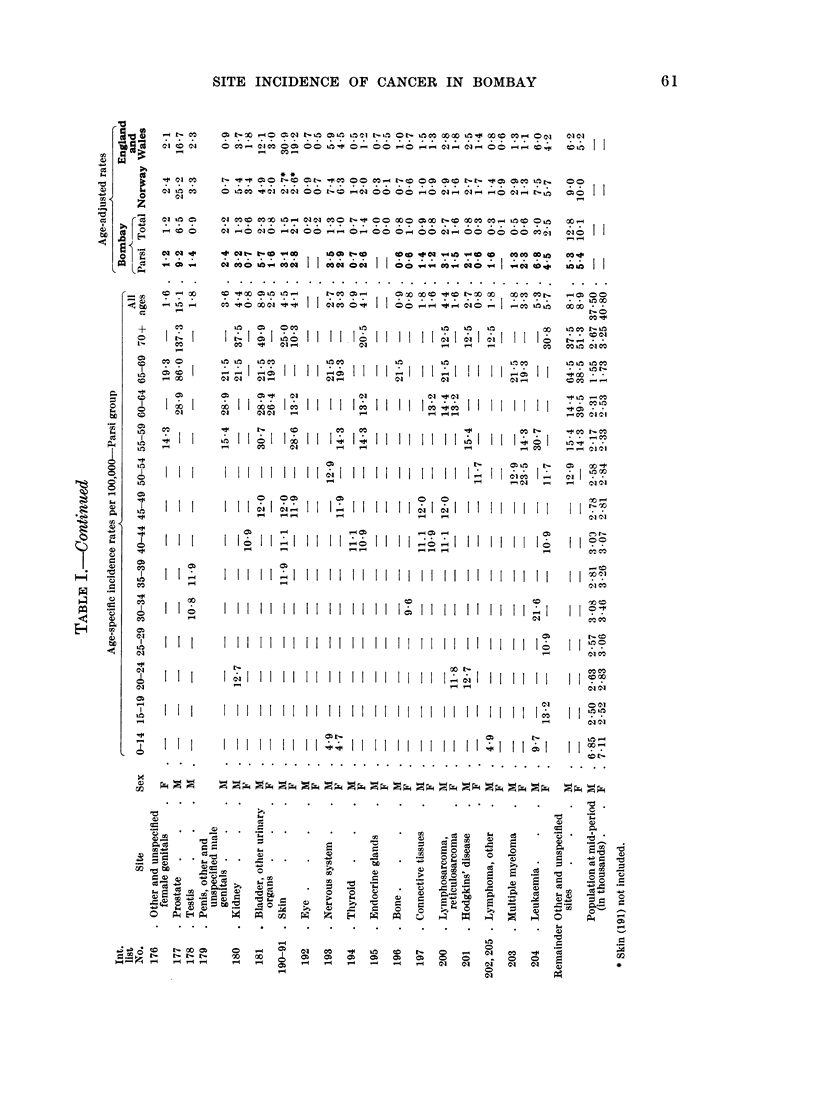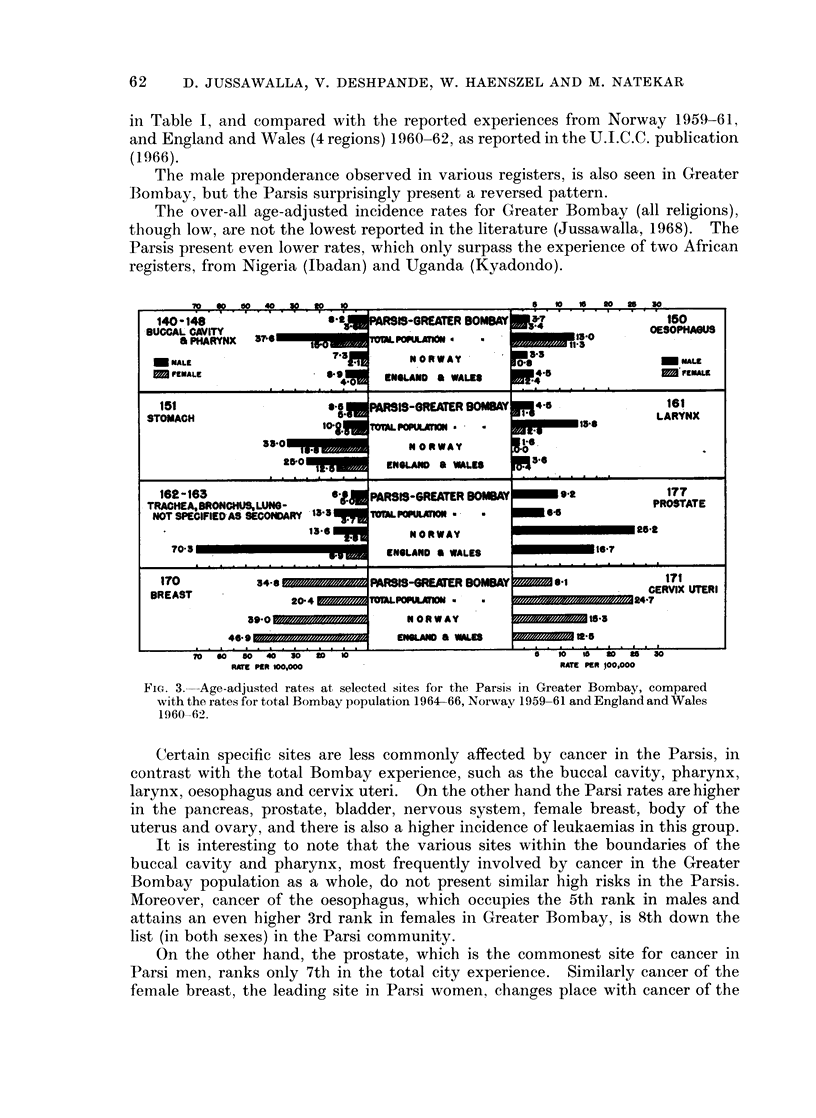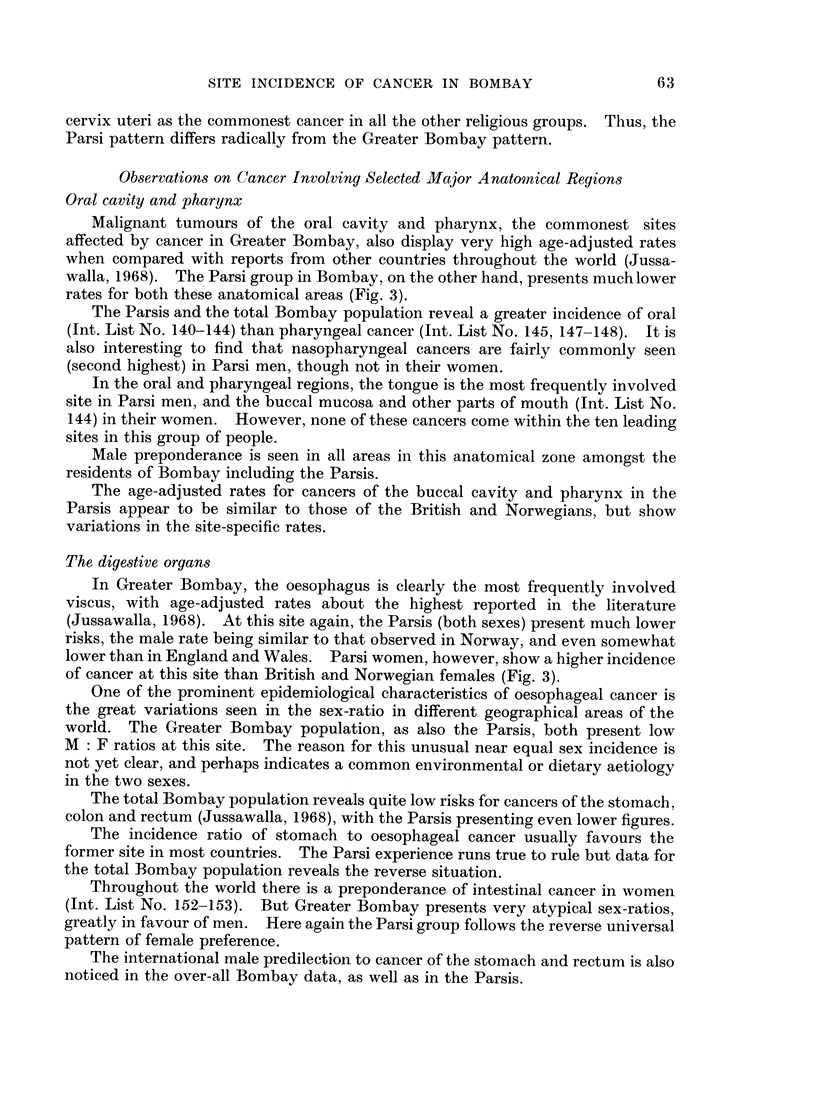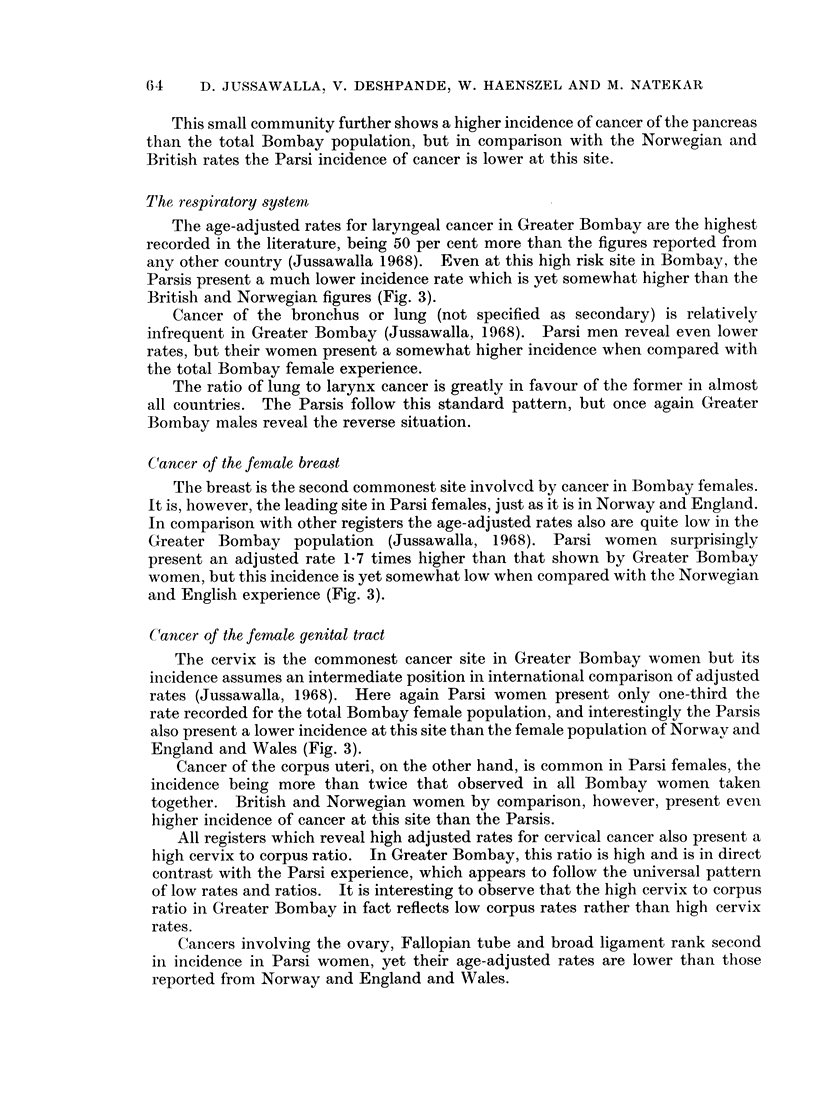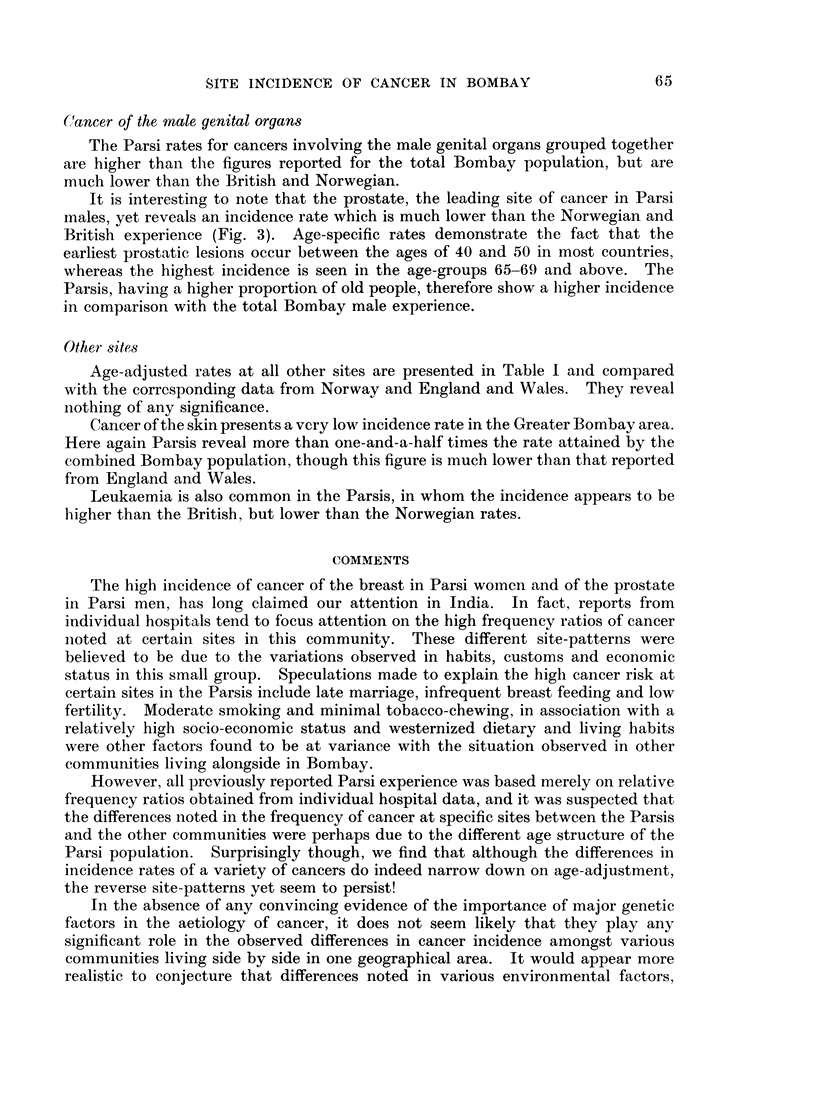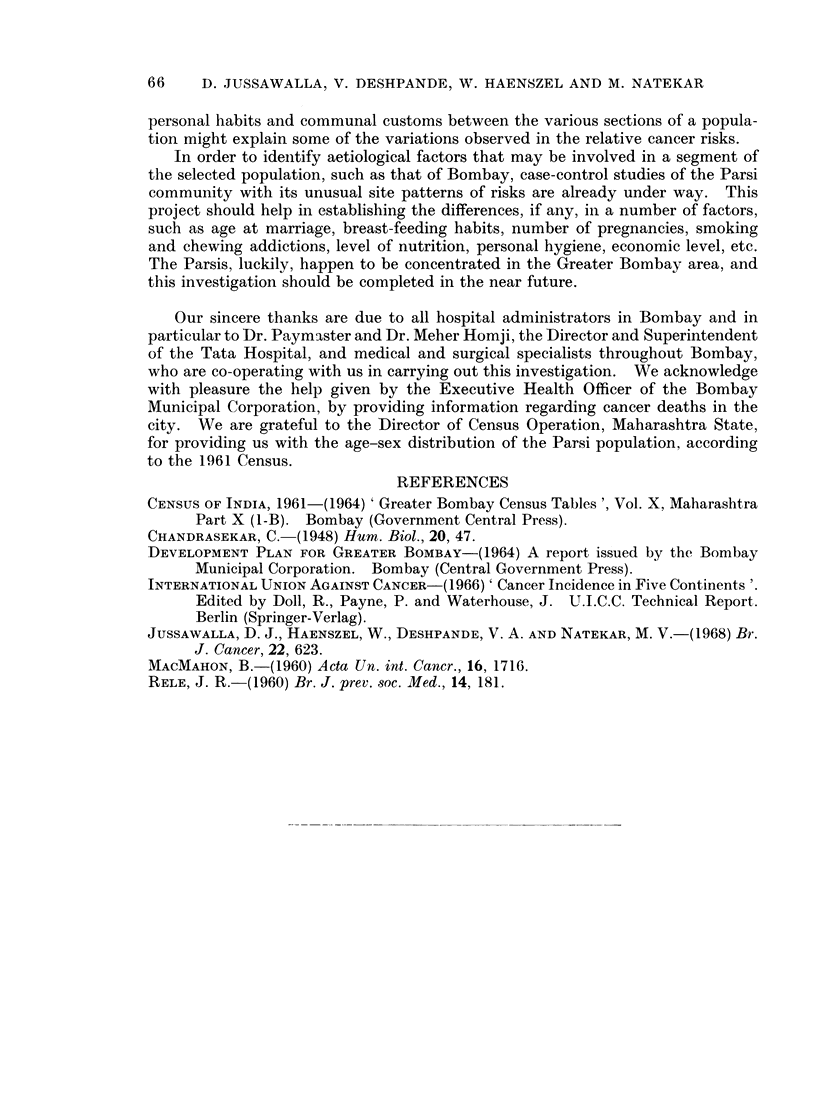# Differences Observed in the Site Incidence of Cancer, Between the Parsi Community and the Total Population of Greater Bombay: A Critical Appraisal

**DOI:** 10.1038/bjc.1970.8

**Published:** 1970-03

**Authors:** D. J. Jussawalla, V. A. Deshpande, W. Haenszel, M. V. Natekar

## Abstract

The Bombay Cancer Registry has been in operation since June 1963 and reliable morbidity data on cancer have since been obtained for the first time in India, from a precisely outlined population base delineated by residential qualifications within strict geographicalboundaries. An attempt has been made to examine the differences noticed in the site-specific cancer risks, between 2 groups of people living in this area—the Parsi community and the total Bombay population. The over-all age adjusted rates for the Parsis were found to be lower than those for the total population and more noticeably, their site-specific risks seem to differ radically from the Greater Bombay pattern. Thus, cancers of the buccal cavity, pharynx, larynx, oesophagus and cervix uteri, which are frequently seen in the total Bombay population, are less commonly observed in the Parsi community. On the other hand the Parsi rates are higher at sites such as the female breast, body of uterus, ovary, prostate and skin and for all leukaemias. Even though the population pyramid of the Parsi community is very different from that of the total population of Bombay, age correction does not change the basic outline of risk patterns noted in the 2 groups. Such site-specific contrasts are believed to be due to differences present in the habits, customs and economic status of the two groups. (A study of the probable aetiological factors of epidemiological importance involved in this segment of the population, is already under way in Bombay, in an effort to identify the reasons for the differences noted in cancer risks at different sites.)


### DIFFERENCES OBSERVED IN THE SITE INCIDENCE OF CANCER, BETWEEN THE PARSI COMMUNITY AND THE TOTAL POPULATION OF GREATER BOMBAY: A CRITICAL APPRAISAL

D. J. JUSSAWALLA, V. A. DESHPANDE, W. HAENSZEL AND M. V. NATEKAR

*From the Bombay Cancer Registry of the Indian Cancer Society, Bombay-12, India, and the Biometry Branch, National Cancer Institute, Bethesda, Maryland, U.S.A.*

Received for publication January 2, 1970

**SUMMARY.—The Bombay Cancer Registry has been in operation since June 1963 and reliable morbidity data on cancer have since been obtained for the first time in India, from a precisely outlined population base delineated by residential qualifications within strict geographical boundaries. An attempt has been made to examine the differences noticed in the site-specific cancer risks, between 2 groups of people living in this area—the Parsi community and the total Bombay population. The over-all age adjusted rates for the Parsis were found to be lower than those for the total population and more noticeably, their site-specific risks seem to differ radically from the Greater Bombay pattern. Thus, cancers of the buccal cavity, pharynx, larynx, oesophagus and cervix uteri, which are frequently seen in the total Bombay population, are less commonly observed in the Parsi community. On the other hand the Parsi rates are higher at sites such as the female breast, body of uterus, ovary, prostate and skin and for all leukaemias. Even though the population pyramid of the Parsi community is very different from that of the total population of Bombay, age correction does not change the basic outline of risk patterns noted in the 2 groups. Such site-specific contrasts are believed to be due to differences present in the habits, customs and economic status of the two groups. (A study of the probable aetiological factors of epidemiological importance involved in this segment of the population, is already under way in Bombay, in an effort to identify the reasons for the differences noted in cancer risks at different sites.)**

GREATER Bombay is a cosmopolitan city with a population of approximately 4,600,000 persons, drawn from every State in the Indian Union. This urban centre thus represents a true cross-section of the heterogeneous peoples of the country. Thinly dispersed in this vast city, a tiny community known as the Parsis, has made significant contributions far beyond its small numbers would perhaps seem to warrant, to make this city the " Urbs primus in Indis ". The Parsis are distinguished by religious, demographic and socio-economic factors and even though living in the same geographical environment, present striking differences from others in the relative frequencies of cancer noted at various sites. A critical appraisal of this situation was thus considered promising, in order to ascertain whether or not these apparent differences could be ascribed to recognizable variations in habits, customs and socio-economic conditions of these people. With this aim in view, we have attempted to investigate the true state of affairs in this small community in Greater Bombay and if possible, to define the magnitude and nature of the variations observed.

*Historical Background of the Parsis*

The Parsis are in fact the sole surviving group of Persian Zoroastrians, who fled their homeland to escape religious persecution by the invading Moslems who finally over-powered the weakened Persian Empire by the middle of the seventh century A.D. These refugees wandered away from Persia in large numbers for almost a century, but only a small group is known to have settled along the west coast of India. What happened to the rest is a mystery. In their pursuit for a better way of life, the Parsis soon moved once again southward (almost 300 years ago) from their early rural settlements along the Gujarat coast, and finally settled in Bombay City in large numbers.

The community is highly inbred and approximately two-thirds of its members, out of a total world population of 120,000, still reside in the Greater Bombay area. The economic status, literacy rate and various habits and customs of these people appear to be at variance with those of other communities residing alongside. This small group is unusually enlightened, enterprising, prosperous and westernized (Chandrasekar, 1948; Rele, 1960).

FIG. 1.—Population for Parsis and all religions, Greater Bombay, 1964–66.

*Area and Population of Greater Bombay*

This survey, undertaken by the Bombay Cancer Registry, is restricted to the residents of Greater Bombay, a densely populated urban centre on the west coast of India, occupying an area of 437·7 sq. km.

The population figures used to compute the incidence rates have been interpolated from the projected population of Greater Bombay (Development Plan for Greater Bombay, 1964). Only those cancer patients have been taken into account who were confirmed as being residents of the metropolis for a minimal period of 1 year.

Special Census of India tables, based on the 1961 data and giving the age-composition of the Parsi population, have been used to estimate their age distribution (personal communication from the Director of Census Operations, Maharashtra State).

The Parsis form but 1·7 per cent of the total number of residents of Greater Bombay, and present an age structure remarkably different from that of the total population of the city (Fig. 1).

### MATERIAL

In this report data obtained from the first three years of survey (1964–66) undertaken by the Bombay Cancer Registry* have been used.

This Registry was established in June 1963, only since when reliable morbidity data on cancer have been obtained in India for the first time, from a precisely defined population base. The details concerning registration and methodology, have been described in a previous publication (Jussawalla *et al.*, 1968).

During the period under review (1964–66), 9703 new cancer cases were detected among the residents of Bombay, of whom only 362 were found to be Parsis.

The percentage of cancer patients having microscopic confirmation of diagnosis, is low for the total Bombay population (Jussawalla *et al.*, 1968), as well as for the Parsi group. This demonstrates the need for increasing the availability of expert pathological services throughout Greater Bombay and it is planned to provide this service in the near future.

A review of the literature on cancer risks in the main religious sects in India shows that such assessments have so far been based entirely on relative frequency data merely obtained from individual hospital records. Variations in the form and frequency of cancer at certain sites have been noted when the Parsi experience was compared with that of the other communities.

The facts presented in this paper, however, are based on a careful analysis of the incidence of cancer in the Parsis of Greater Bombay, and represent a direct estimation of the true level of cancer risk (incidence) in this group as compared with that of the total city population.

### *Cancer Incidence Rates*

A vast amount of effort seems to have been devoted to the study of cancer frequency in the white and non-white populations of the United States. These studies have revealed significant differences in cancer incidence between these 2 ethnic groups and have proved useful in elucidating a number of aetiological factors responsible for the occurrence of cancer at specific sites. There have been, however, very few enquiries undertaken elsewhere to estimate the difference in cancer risks between ethnic sub-groups and between various religious sects living together in one geographical area (MacMahon, 1960).

We have attempted here to examine the cancer incidence rates at various anatomical sites in the different religious groups residing in Greater Bombay in order to obtain evidence of variations, if present. Although the population of Bombay consists of a number of differing religious sects, data obtained from the whole of the metropolitan area tends to reflect predominantly merely the Hindu experience, because of the overwhelming numbers of these people. The individual Hindu, Muslim and Christian contrasts are beyond the scope of this paper, which

* The Registry is a unit of the Indian Cancer Society at Bombay, and is supported in part by the National Cancer Institute at Bethesda, U.S.A., through research grant NIH–01–006–1.

is restricted to the differences noted in site-specific cancer risks between the Parsis and the total Bombay population.

The sex and age-specific incidence rates are presented in Table I for the Parsi residents of Greater Bombay, whereas the age-specific cancer incidence rates at all sites in this group are compared with those of the total population of Bombay in Fig. 2.

FIG. 2.—Age-specific cancer incidence rates per 100,000 population for all religions and Parsis, Greater Bombay, 1964–66.

The variations in the age-specific incidence rates are due to the smallness of the sample of some age-groups but, on the whole, incidence rates tend to follow the general pattern of increase with age for both the populations studied.

*Age-adjusted rates*

For adequate comparison, all cancer incidence rates have been adjusted according to the world standard population, as suggested by Doll *et al.* (International Union against Cancer, 1966). Age-adjusted rates computed for the Parsi community and for the Bombay population taken as a whole are presented

TABLE I.—*Age-specific Cancer Incidence Rates Among Parsis in Greater Bombay by Sex and Primary Site*, 1964–66, *and Age-adjusted Rates for Greater Bombay* 1964–66, *Norway* 1959–61, *England and Wales* (4 *Regions*), 1960–62

| | | | Age-specific incidence rates per 100,000—Parsi group | | | | | | | | | | | | | | Age-adjusted rates | | | |
|---|---|---|---|---|---|---|---|---|---|---|---|---|---|---|---|---|---|---|---|---|
| | | | | | | | | | | | | | | | | | Bombay | | | England and |
| Int. list No. | Site | Sex | 0–14 | 15–19 | 20–24 | 25–29 | 30–34 | 35–39 | 40–44 | 45–49 | 50–54 | 55–59 | 60–64 | 65–69 | 70+ | All ages | Parsi | Total | Norway | Wales |
| 140–205 | All sites | M | 19·5 | — | 25·3 | 13·0 | 32·5 | 47·4 | 55·6 | 107·9 | 116·3 | 199·7 | 274·2 | 602·2 | 699·1 | 136·0 | **96·1** | 139·5 | 165·0 | 246·0 |
| | | F | 4·7 | 26·5 | 11·8 | 10·9 | 48·2 | 30·7 | 152·0 | 177·9 | 270·0 | 343·3 | 434·8 | 597·3 | 574·4 | 170·8 | **115·3** | 131·1 | 162·2 | 185·9 |
| 140 | Lip | M | — | — | — | — | — | — | — | — | — | — | — | — | 12·5 | 0·9 | **0·5** | 0·4 | 3·5 | 2·0 |
| | | F | — | — | — | — | — | — | — | — | — | — | — | — | — | — | — | 0·4 | 0·1 | 0·3 |
| 141 | Tongue | M | — | — | — | — | — | 11·9 | — | 12·0 | — | — | — | 21·5 | — | 2·7 | **2·1** | 14·0 | 0·8 | 1·5 |
| | | F | — | — | — | — | — | — | — | — | — | 14·3 | 13·2 | — | 10·3 | 2·5 | **1·5** | 3·7 | 0·5 | 0·6 |
| 142 | Salivary glands | M | — | — | — | — | — | — | — | — | — | — | — | — | — | — | — | 0·1 | 0·4 | 1·1 |
| | | F | — | — | — | — | — | — | — | — | — | — | — | — | — | — | — | 0·1 | 0·3 | 1·2 |
| 143–44 | Mouth, other and unspecified | M | — | — | — | — | — | — | — | — | 12·9 | — | — | — | 25·0 | 2·7 | **1·6** | 6·5 | 1·3 | 1·7 |
| | | F | — | — | — | — | — | — | — | — | — | 14·3 | — | 19·3 | 10·3 | 2·5 | **1·6** | 5·9 | 0·6 | 0·6 |
| 146 | Nasopharynx | M | — | — | — | — | — | 11·9 | — | — | — | — | 14·4 | — | 12·5 | 2·7 | **1·8** | 0·6 | 0·3 | 0·5 |
| | | F | — | — | — | — | — | — | — | — | — | — | — | — | — | — | — | 0·5 | 0·1 | 0·2 |
| 145, 147–48 | Pharynx | M | — | — | — | — | — | — | — | — | — | — | 14·4 | 21·5 | 25·0 | 3·6 | **2·2** | 16·0 | 1·0 | 2·1 |
| | | F | — | — | — | — | — | — | — | — | — | — | 13·2 | — | — | 0·8 | **0·5** | 4·4 | 0·5 | 1·1 |
| 150 | Oesophagus | M | — | — | — | — | — | — | — | 12·0 | 12·9 | — | — | 43·0 | 25·0 | 5·3 | **3·7** | 13·0 | 3·3 | 4·5 |
| | | F | — | — | — | — | — | — | — | 11·9 | — | — | 39·5 | 38·5 | — | 4·9 | **3·4** | 11·3 | 0·8 | 2·4 |
| 151 | Stomach | M | — | — | — | — | — | — | — | — | 12·9 | 15·4 | 57·7 | 64·5 | 74·9 | 13·3 | **8·5** | 10·0 | 33·0 | 24·8 |
| | | F | — | — | — | — | — | — | 21·7 | — | — | 14·3 | 13·2 | 38·5 | 51·3 | 9·0 | **5·6** | 6·5 | 18·8 | 12·4 |
| 152–53 | Intestine | M | — | — | — | — | — | — | — | — | — | 15·4 | 14·4 | — | 37·5 | 4·4 | **2·7** | 4·2 | 10·9 | 14·5 |
| | | F | — | — | — | — | — | — | — | 11·9 | 23·5 | 14·3 | — | 38·5 | 30·8 | 7·4 | **4·8** | 3·8 | 10·5 | 14·9 |
| 154 | Rectum | M | — | — | — | — | — | — | — | — | 12·9 | 15·4 | 14·4 | 43·0 | 12·5 | 5·3 | **3·6** | 4·3 | 6·5 | 13·1 |
| | | F | — | — | — | — | — | — | — | — | — | 42·9 | — | 19·3 | 10·3 | 4·1 | **2·7** | 3·5 | 4·2 | 7·8 |
| 155 | Biliary passage, liver-primary | M | — | — | — | — | — | — | — | — | 12·9 | — | — | — | — | 0·9 | **0·6** | 1·1 | 2·0 | 2·2 |
| | | F | — | — | — | — | — | — | — | — | — | — | — | 19·3 | — | 0·8 | **0·6** | 0·9 | 2·1 | 2·0 |
| 157 | Pancreas | M | — | — | — | — | — | — | 11·1 | — | 12·9 | — | 14·4 | 64·5 | 25·0 | 7·1 | **4·8** | 2·0 | 5·9 | 6·6 |
| | | F | — | — | — | — | — | — | — | — | — | 14·3 | — | 19·3 | 10·3 | 2·5 | **1·6** | 0·9 | 3·8 | 3·9 |
| 160 | Nose, nasal cavities, accessory sinuses | M | — | — | — | — | — | — | — | — | — | — | — | — | — | — | — | 1·4 | 0·8 | 0·8 |
| | | F | — | — | — | — | — | — | — | — | — | — | — | 19·3 | 10·3 | 1·6 | **1·0** | 1·0 | 0·4 | 0·5 |
| 161 | Larynx | M | — | — | — | — | — | — | — | 12·0 | — | 15·4 | — | 21·5 | 62·4 | 7·1 | **4·5** | 13·8 | 1·6 | 3·6 |
| | | F | — | — | — | — | — | — | — | — | 11·7 | — | 26·4 | — | — | 2·5 | **1·6** | 2·8 | 0·0 | 0·4 |
| 162–63 | Trachea, bronchus, lung —not specified as secondary | M | — | — | — | 13·0 | — | — | — | 24·0 | — | 30·7 | 14·4 | 21·5 | 49·9 | 9·8 | **6·8** | 13·3 | 13·6 | 70·3 |
| | | F | — | — | — | — | — | — | — | 11·9 | 23·5 | — | 13·2 | 19·3 | 51·3 | 8·2 | **5·0** | 3·7 | 2·8 | 8·9 |
| 170 | Breast | M | — | — | — | — | — | — | — | — | — | — | 14·4 | — | 12·5 | 1·8 | **1·1** | 0·1 | 0·3 | 0·5 |
| | | F | — | — | — | — | 28·9 | — | 76·0 | 71·2 | 140·8 | 85·8 | 118·6 | 96·3 | 153·8 | 51·5 | **34·8** | 20·4 | 39·0 | 46·9 |
| 171 | Cervix uteri | F | — | — | — | — | — | — | — | — | — | 42·9 | 26·4 | 96·3 | 61·5 | 13·1 | **8·1** | 24·7 | 15·3 | 12·5 |
| 172 | Corpus uteri | F | — | — | — | — | 9·6 | — | 10·9 | 11·9 | 11·7 | — | — | 19·3 | — | 4·1 | **3·1** | 1·4 | 7·7 | 9·1 |
| 173 | Chorionepithelioma uterus | F | — | — | — | — | — | — | — | — | — | — | — | — | — | — | — | 0·2 | 0·2 | 0·7 |
| 174 | Uterus, other and unspecified | F | — | — | — | — | — | — | — | — | — | — | 13·2 | 19·3 | 41·0 | 4·9 | **2·7** | 4·8 | 1·0 | — |
| 175 | Ovary, tube, broad ligament | F | — | 13·2 | — | — | — | 30·7 | — | 35·6 | 11·7 | — | 39·5 | 19·3 | 20·5 | 11·4 | **8·7** | 6·1 | 11·5 | 10·5 |

TABLE I.—*Continued*

| Int. list No. | Site | Sex | Age-specific incidence rates per 100,000—Parsi group: 0–14 | 15–19 | 20–24 | 25–29 | 30–34 | 35–39 | 40–44 | 45–49 | 50–54 | 55–59 | 60–64 | 65–69 | 70+ | All ages | Age-adjusted rates: Bombay Parsi | Bombay Total | Norway | England and Wales |
|---|---|---|---|---|---|---|---|---|---|---|---|---|---|---|---|---|---|---|---|---|
| 176 | Other and unspecified female genitals | F | — | — | — | — | — | — | — | — | — | 14·3 | — | 19·3 | — | 1·6 | 1·2 | 1·2 | 2·4 | 2·1 |
| 177 | Prostate | M | — | — | — | — | — | — | — | — | — | — | 28·9 | 86·0 | 137·3 | 15·1 | 9·2 | 6·5 | 25·2 | 16·7 |
| 178 | Testis | M | — | — | — | — | 10·8 | 11·9 | — | — | — | — | — | — | — | 1·8 | 1·4 | 0·9 | 3·3 | 2·3 |
| 179 | Penis, other and unspecified male genitals | M | — | — | — | — | — | — | — | — | — | 15·4 | 28·9 | 21·5 | — | 3·6 | 2·4 | 2·2 | 0·7 | 0·9 |
| 180 | Kidney | M | — | — | 12·7 | — | — | — | — | — | — | — | — | 21·5 | 37·5 | 4·4 | 3·2 | 1·3 | 5·4 | 3·7 |
| | | F | — | — | — | — | — | — | 10·9 | — | — | — | — | — | — | 0·8 | 0·7 | 0·6 | 3·4 | 1·8 |
| 181 | Bladder, other urinary organs | M | — | — | — | — | — | — | — | 12·0 | — | 30·7 | 28·9 | 21·5 | 49·9 | 8·9 | 5·7 | 2·3 | 4·9 | 12·1 |
| | | F | — | — | — | — | — | — | — | — | — | — | 26·4 | 19·3 | — | 2·5 | 1·6 | 0·8 | 2·0 | 3·0 |
| 190–91 | Skin | M | — | — | — | — | — | 11·9 | 11·1 | 12·0 | — | — | — | — | 25·0 | 4·5 | 3·1 | 1·5 | 2·7* | 30·9 |
| | | F | — | — | — | — | — | — | — | 11·9 | — | 28·6 | 13·2 | — | 10·3 | 4·1 | 2·8 | 2·1 | 2·6* | 19·2 |
| 192 | Eye | M | — | — | — | — | — | — | — | — | — | — | — | — | — | — | — | 0·2 | 0·9 | 0·7 |
| | | F | — | — | — | — | — | — | — | — | — | — | — | — | — | — | — | 0·2 | 0·7 | 0·5 |
| 193 | Nervous system | M | 4·9 | — | — | — | — | — | — | — | 12·9 | — | — | 21·5 | — | 2·7 | 3·5 | 1·3 | 7·4 | 5·9 |
| | | F | 4·7 | — | — | — | — | — | — | 11·9 | — | 14·3 | — | 19·3 | — | 3·3 | 2·9 | 1·0 | 6·3 | 4·5 |
| 194 | Thyroid | M | — | — | — | — | — | — | 11·1 | — | — | — | — | — | — | 0·9 | 0·7 | 0·7 | 1·0 | 0·5 |
| | | F | — | — | — | — | — | — | 10·9 | — | — | 14·3 | 13·2 | — | 20·5 | 4·1 | 2·6 | 1·4 | 2·0 | 1·2 |
| 195 | Endocrine glands | M | — | — | — | — | — | — | — | — | — | — | — | — | — | — | — | 0·0 | 0·3 | 0·7 |
| | | F | — | — | — | — | — | — | — | — | — | — | — | — | — | — | — | 0·0 | 0·1 | 0·5 |
| 196 | Bone | M | — | — | — | — | — | — | — | — | — | — | — | 21·5 | — | 0·9 | 0·6 | 0·8 | 0·7 | 1·0 |
| | | F | — | — | — | — | 9·6 | — | — | — | — | — | — | — | — | 0·8 | 0·6 | 1·0 | 0·6 | 0·7 |
| 197 | Connective tissues | M | — | — | — | — | — | — | 11.1 | 12·0 | — | — | — | — | — | 1·8 | 1·4 | 0·9 | 1·0 | 1·5 |
| | | F | — | — | — | — | — | — | 10·9 | — | — | — | 13·2 | — | — | 1·6 | 1·2 | 0·8 | 0·9 | 1·3 |
| 200 | Lymphosarcoma, reticulosarcoma | M | — | — | — | — | — | — | 11·1 | 12·0 | — | — | 14·4 | 21·5 | 12·5 | 4·4 | 3·1 | 2·7 | 2·9 | 2·8 |
| | | F | — | — | 11·8 | — | — | — | — | — | — | — | 13·2 | — | — | 1·6 | 1·5 | 1·6 | 1·6 | 1·8 |
| 201 | Hodgkins' disease | M | — | — | 12·7 | — | — | — | — | — | — | 15·4 | — | — | 12·5 | 2·7 | 2·1 | 0·8 | 2·7 | 2·5 |
| | | F | — | — | — | — | — | — | — | — | 11·7 | — | — | — | — | 0·8 | 0·6 | 0·3 | 1·7 | 1·4 |
| 202, 205 | Lymphoma, other | M | 4·9 | — | — | — | — | — | — | — | — | — | — | — | 12·5 | 1·8 | 1·6 | 0·3 | 1·4 | 0·8 |
| | | F | — | — | — | — | — | — | — | — | — | — | — | — | — | — | — | 0·1 | 0·9 | 0·6 |
| 203 | Multiple myeloma | M | — | — | — | — | — | — | — | — | 12·9 | — | — | 21·5 | — | 1·8 | 1·3 | 0·5 | 2·9 | 1·3 |
| | | F | — | — | — | — | — | — | — | — | 23·5 | 14·3 | — | 19·3 | — | 3·3 | 2·3 | 0·6 | 1·3 | 1·1 |
| 204 | Leukaemia | M | 9·7 | — | — | — | 21·6 | — | — | — | — | 30·7 | — | — | — | 5·3 | 6·8 | 3·0 | 7·5 | 6·0 |
| | | F | — | 13·2 | — | 10·9 | — | — | 10·9 | — | 11·7 | — | — | — | 30·8 | 5·7 | 4·5 | 2·5 | 5·7 | 4·2 |
| Remainder | Other and unspecified sites | M | — | — | — | — | — | — | — | — | 12·9 | 15·4 | 14·4 | 64·5 | 37·5 | 8·1 | 5·3 | 12·8 | 9·0 | 6·2 |
| | | F | — | — | — | — | — | — | — | — | — | 14·3 | 39·5 | 38·5 | 51·3 | 8·9 | 5·4 | 10·1 | 10·0 | 5·2 |
| | Population at mid-period (in thousands) | M | 6·85 | 2·50 | 2·63 | 2·57 | 3·08 | 2·81 | 3·00 | 2·78 | 2·58 | 2·17 | 2·31 | 1·55 | 2·67 | 37·50 | — | — | — | — |
| | | F | 7·11 | 2·52 | 2·83 | 3·06 | 3·46 | 3·26 | 3·07 | 2·81 | 2·84 | 2·33 | 2·53 | 1·73 | 3·25 | 40·80 | — | — | — | — |

* Skin (191) not included.

in Table I, and compared with the reported experiences from Norway 1959–61, and England and Wales (4 regions) 1960–62, as reported in the U.I.C.C. publication (1966).

The male preponderance observed in various registers, is also seen in Greater Bombay, but the Parsis surprisingly present a reversed pattern.

The over-all age-adjusted incidence rates for Greater Bombay (all religions), though low, are not the lowest reported in the literature (Jussawalla, 1968). The Parsis present even lower rates, which only surpass the experience of two African registers, from Nigeria (Ibadan) and Uganda (Kyadondo).

FIG. 3.—Age-adjusted rates at selected sites for the Parsis in Greater Bombay, compared with the rates for total Bombay population 1964–66, Norway 1959–61 and England and Wales 1960–62.

Certain specific sites are less commonly affected by cancer in the Parsis, in contrast with the total Bombay experience, such as the buccal cavity, pharynx, larynx, oesophagus and cervix uteri. On the other hand the Parsi rates are higher in the pancreas, prostate, bladder, nervous system, female breast, body of the uterus and ovary, and there is also a higher incidence of leukaemias in this group.

It is interesting to note that the various sites within the boundaries of the buccal cavity and pharynx, most frequently involved by cancer in the Greater Bombay population as a whole, do not present similar high risks in the Parsis. Moreover, cancer of the oesophagus, which occupies the 5th rank in males and attains an even higher 3rd rank in females in Greater Bombay, is 8th down the list (in both sexes) in the Parsi community.

On the other hand, the prostate, which is the commonest site for cancer in Parsi men, ranks only 7th in the total city experience. Similarly cancer of the female breast, the leading site in Parsi women, changes place with cancer of the cervix uteri as the commonest cancer in all the other religious groups. Thus, the Parsi pattern differs radically from the Greater Bombay pattern.

*Observations on Cancer Involving Selected Major Anatomical Regions*

*Oral cavity and pharynx*

Malignant tumours of the oral cavity and pharynx, the commonest sites affected by cancer in Greater Bombay, also display very high age-adjusted rates when compared with reports from other countries throughout the world (Jussawalla, 1968). The Parsi group in Bombay, on the other hand, presents much lower rates for both these anatomical areas (Fig. 3).

The Parsis and the total Bombay population reveal a greater incidence of oral (Int. List No. 140–144) than pharyngeal cancer (Int. List No. 145, 147–148). It is also interesting to find that nasopharyngeal cancers are fairly commonly seen (second highest) in Parsi men, though not in their women.

In the oral and pharyngeal regions, the tongue is the most frequently involved site in Parsi men, and the buccal mucosa and other parts of mouth (Int. List No. 144) in their women. However, none of these cancers come within the ten leading sites in this group of people.

Male preponderance is seen in all areas in this anatomical zone amongst the residents of Bombay including the Parsis.

The age-adjusted rates for cancers of the buccal cavity and pharynx in the Parsis appear to be similar to those of the British and Norwegians, but show variations in the site-specific rates.

*The digestive organs*

In Greater Bombay, the oesophagus is clearly the most frequently involved viscus, with age-adjusted rates about the highest reported in the literature (Jussawalla, 1968). At this site again, the Parsis (both sexes) present much lower risks, the male rate being similar to that observed in Norway, and even somewhat lower than in England and Wales. Parsi women, however, show a higher incidence of cancer at this site than British and Norwegian females (Fig. 3).

One of the prominent epidemiological characteristics of oesophageal cancer is the great variations seen in the sex-ratio in different geographical areas of the world. The Greater Bombay population, as also the Parsis, both present low M : F ratios at this site. The reason for this unusual near equal sex incidence is not yet clear, and perhaps indicates a common environmental or dietary aetiology in the two sexes.

The total Bombay population reveals quite low risks for cancers of the stomach, colon and rectum (Jussawalla, 1968), with the Parsis presenting even lower figures.

The incidence ratio of stomach to oesophageal cancer usually favours the former site in most countries. The Parsi experience runs true to rule but data for the total Bombay population reveals the reverse situation.

Throughout the world there is a preponderance of intestinal cancer in women (Int. List No. 152–153). But Greater Bombay presents very atypical sex-ratios, greatly in favour of men. Here again the Parsi group follows the reverse universal pattern of female preference.

The international male predilection to cancer of the stomach and rectum is also noticed in the over-all Bombay data, as well as in the Parsis.

This small community further shows a higher incidence of cancer of the pancreas than the total Bombay population, but in comparison with the Norwegian and British rates the Parsi incidence of cancer is lower at this site.

*The respiratory system*

The age-adjusted rates for laryngeal cancer in Greater Bombay are the highest recorded in the literature, being 50 per cent more than the figures reported from any other country (Jussawalla 1968). Even at this high risk site in Bombay, the Parsis present a much lower incidence rate which is yet somewhat higher than the British and Norwegian figures (Fig. 3).

Cancer of the bronchus or lung (not specified as secondary) is relatively infrequent in Greater Bombay (Jussawalla, 1968). Parsi men reveal even lower rates, but their women present a somewhat higher incidence when compared with the total Bombay female experience.

The ratio of lung to larynx cancer is greatly in favour of the former in almost all countries. The Parsis follow this standard pattern, but once again Greater Bombay males reveal the reverse situation.

*Cancer of the female breast*

The breast is the second commonest site involved by cancer in Bombay females. It is, however, the leading site in Parsi females, just as it is in Norway and England. In comparison with other registers the age-adjusted rates also are quite low in the Greater Bombay population (Jussawalla, 1968). Parsi women surprisingly present an adjusted rate 1·7 times higher than that shown by Greater Bombay women, but this incidence is yet somewhat low when compared with the Norwegian and English experience (Fig. 3).

*Cancer of the female genital tract*

The cervix is the commonest cancer site in Greater Bombay women but its incidence assumes an intermediate position in international comparison of adjusted rates (Jussawalla, 1968). Here again Parsi women present only one-third the rate recorded for the total Bombay female population, and interestingly the Parsis also present a lower incidence at this site than the female population of Norway and England and Wales (Fig. 3).

Cancer of the corpus uteri, on the other hand, is common in Parsi females, the incidence being more than twice that observed in all Bombay women taken together. British and Norwegian women by comparison, however, present even higher incidence of cancer at this site than the Parsis.

All registers which reveal high adjusted rates for cervical cancer also present a high cervix to corpus ratio. In Greater Bombay, this ratio is high and is in direct contrast with the Parsi experience, which appears to follow the universal pattern of low rates and ratios. It is interesting to observe that the high cervix to corpus ratio in Greater Bombay in fact reflects low corpus rates rather than high cervix rates.

Cancers involving the ovary, Fallopian tube and broad ligament rank second in incidence in Parsi women, yet their age-adjusted rates are lower than those reported from Norway and England and Wales.

*Cancer of the male genital organs*

The Parsi rates for cancers involving the male genital organs grouped together are higher than the figures reported for the total Bombay population, but are much lower than the British and Norwegian.

It is interesting to note that the prostate, the leading site of cancer in Parsi males, yet reveals an incidence rate which is much lower than the Norwegian and British experience (Fig. 3). Age-specific rates demonstrate the fact that the earliest prostatic lesions occur between the ages of 40 and 50 in most countries, whereas the highest incidence is seen in the age-groups 65–69 and above. The Parsis, having a higher proportion of old people, therefore show a higher incidence in comparison with the total Bombay male experience.

*Other sites*

Age-adjusted rates at all other sites are presented in Table I and compared with the corresponding data from Norway and England and Wales. They reveal nothing of any significance.

Cancer of the skin presents a very low incidence rate in the Greater Bombay area. Here again Parsis reveal more than one-and-a-half times the rate attained by the combined Bombay population, though this figure is much lower than that reported from England and Wales.

Leukaemia is also common in the Parsis, in whom the incidence appears to be higher than the British, but lower than the Norwegian rates.

### COMMENTS

The high incidence of cancer of the breast in Parsi women and of the prostate in Parsi men, has long claimed our attention in India. In fact, reports from individual hospitals tend to focus attention on the high frequency ratios of cancer noted at certain sites in this community. These different site-patterns were believed to be due to the variations observed in habits, customs and economic status in this small group. Speculations made to explain the high cancer risk at certain sites in the Parsis include late marriage, infrequent breast feeding and low fertility. Moderate smoking and minimal tobacco-chewing, in association with a relatively high socio-economic status and westernized dietary and living habits were other factors found to be at variance with the situation observed in other communities living alongside in Bombay.

However, all previously reported Parsi experience was based merely on relative frequency ratios obtained from individual hospital data, and it was suspected that the differences noted in the frequency of cancer at specific sites between the Parsis and the other communities were perhaps due to the different age structure of the Parsi population. Surprisingly though, we find that although the differences in incidence rates of a variety of cancers do indeed narrow down on age-adjustment, the reverse site-patterns yet seem to persist!

In the absence of any convincing evidence of the importance of major genetic factors in the aetiology of cancer, it does not seem likely that they play any significant role in the observed differences in cancer incidence amongst various communities living side by side in one geographical area. It would appear more realistic to conjecture that differences noted in various environmental factors, personal habits and communal customs between the various sections of a population might explain some of the variations observed in the relative cancer risks.

In order to identify aetiological factors that may be involved in a segment of the selected population, such as that of Bombay, case-control studies of the Parsi community with its unusual site patterns of risks are already under way. This project should help in establishing the differences, if any, in a number of factors, such as age at marriage, breast-feeding habits, number of pregnancies, smoking and chewing addictions, level of nutrition, personal hygiene, economic level, etc. The Parsis, luckily, happen to be concentrated in the Greater Bombay area, and this investigation should be completed in the near future.

Our sincere thanks are due to all hospital administrators in Bombay and in particular to Dr. Paymaster and Dr. Meher Homji, the Director and Superintendent of the Tata Hospital, and medical and surgical specialists throughout Bombay, who are co-operating with us in carrying out this investigation. We acknowledge with pleasure the help given by the Executive Health Officer of the Bombay Municipal Corporation, by providing information regarding cancer deaths in the city. We are grateful to the Director of Census Operation, Maharashtra State, for providing us with the age-sex distribution of the Parsi population, according to the 1961 Census.

### REFERENCES

CENSUS OF INDIA, 1961—(1964) ' Greater Bombay Census Tables ', Vol. X, Maharashtra Part X (1-B). Bombay (Government Central Press).

CHANDRASEKAR, C.—(1948) *Hum. Biol.*, **20**, 47.

DEVELOPMENT PLAN FOR GREATER BOMBAY—(1964) A report issued by the Bombay Municipal Corporation. Bombay (Central Government Press).

INTERNATIONAL UNION AGAINST CANCER—(1966) ' Cancer Incidence in Five Continents '. Edited by Doll, R., Payne, P. and Waterhouse, J. U.I.C.C. Technical Report. Berlin (Springer-Verlag).

JUSSAWALLA, D. J., HAENSZEL, W., DESHPANDE, V. A. AND NATEKAR, M. V.—(1968) *Br. J. Cancer*, **22**, 623.

MACMAHON, B.—(1960) *Acta Un. int. Cancr.*, **16**, 1716.

RELE, J. R.—(1960) *Br. J. prev. soc. Med.*, **14**, 181.